# Word Learning in Children With Developmental Language Disorder: The Use of Retrieval Practice During Shared Book Reading

**DOI:** 10.1044/2025_JSLHR-24-00809

**Published:** 2025-06-16

**Authors:** Sofia Souto, Laurence B. Leonard, Patricia Deevy, Sharon L. Christ, Jeffrey D. Karpicke, Mariel L. Schroeder

**Affiliations:** a Department of Speech, Language, and Hearing Sciences, Butler University, Indianapolis, IN; b Department of Speech, Language, and Hearing Sciences, Purdue University, West Lafayette, IN; c Department of Human Development and Family Science, Purdue University, West Lafayette, IN; d Department of Psychological Sciences, Purdue University, West Lafayette, IN

## Abstract

**Purpose:**

Children with developmental language disorder (DLD) benefit from the inclusion of retrieval practice during word learning. However, most studies reporting this positive effect have been conducted in controlled laboratory conditions. In this study, we take a step toward real-world application by matching the design details of a previous laboratory study and inserting them in a shared book reading activity.

**Method:**

Thirteen children with DLD (*M*_age_ = 59.13 months) and 14 children with typical language development (TD; *M*_age_ = 57.07 months) learned eight novel words presented in two illustrated children's books. Half of the novel words appeared in a repeated spaced retrieval (RSR) condition, and half appeared in a repeated study (RS) condition. The children learned both the novel word forms (e.g., /bog/) and their arbitrarily assigned “meanings” (e.g., “likes rain”) in two learning sessions. Five minutes after the second learning session and 1 week later, the children's ability to learn the novel words was assessed.

**Results:**

Both groups of children showed better recall of the novel words in the RSR condition than in the RS condition. This was true for both the novel word forms and their meanings. Scores on a recognition test did not show a difference between the two conditions. The children with TD performed at a higher level than the children with DLD on the word form recall and recognition tests. Both groups showed only a slight decline in word form recall after 1 week. There were no interactions.

**Conclusions:**

The results indicate that incorporation of retrieval practice into shared book reading activities can produce benefits to children's word learning. These findings should encourage future retrieval practice studies with ever closer approximations to the everyday shared book reading experiences of children.

How many words do children know? The answer to the question depends on many different factors, the most obvious being the particular age of the children. For example, it has been estimated that beginning at 24 months, children acquire about 10 words per day and reach approximately 14,000 words by 6 years of age (Carey, 1978; Templin, 1957; see Clark, 1993). However, these word counts, like any other, depend on decisions made about how knowing a word is defined. Factors to consider include whether measurement involves receptive vocabulary or expressive vocabulary and whether vocabulary counts are limited to lemmas (e.g., “play”) or also include inflections (“played”) and derivations (“playful”). In addition, the depth of a child's knowledge of each word must be considered.

Whatever the criteria used, it appears that one group of children—children with developmental language disorder (DLD)—will consistently fall behind their same-age peers with typical language development (TD). This is seemingly true across all ages of childhood and beyond (Rice & Hoffman, 2015), whether vocabulary is measured in terms of depth or breadth of knowledge (McGregor et al., 2013) and regardless of whether word learning is measured in terms of accumulated knowledge on standardized tests (Gray et al., 1999) or a dynamic measure such as the amount of exposure or time required to learn a set of novel words in a laboratory setting (Alt, 2011; Kan & Windsor, 2010; Rice et al., 1994).

These children's limitations in word learning are not likely isolated from other challenges the children have with language. For example, some minimal inventory of words appears to be necessary to promote early grammatical development (Bates & Goodman, 1999). Once grammar emerges, much of word learning (especially verb learning) depends on the children's ability to develop hypotheses about the words' meanings based in part on the sentence frames in which the words appear (Gleitman et al., 2005). A limited vocabulary, then, can slow the development of grammar, and difficulties with grammatical processing can, in turn, slow the subsequent growth of vocabulary. These vocabulary weaknesses can spell trouble for the children's near- and longer-term future. For example, vocabulary knowledge is an important predictor of later achievements, including reading skill (e.g., Muter et al., 2004; Nation et al., 2022)—a vulnerable area in DLD (Catts et al., 2002; Snowling & Hulme, 2021; Ziegenfusz et al., 2022).

## Retrieval Practice and Word Learning

Numerous studies have been aimed at facilitating the vocabulary growth of children with DLD (Law et al., 2003). In recent years, several research teams have asked whether these children's word learning weaknesses might be strengthened if intervention activities included the use of “retrieval practice.” This term refers to the act of trying to recall information just studied throughout the learning phase rather than postponing recall attempts until the end of the learning period. The crucial insight behind retrieval practice is that attempting to recall information during the learning phase actually creates new learning. These attempts are not simply passive events that reflect how much has already been learned. This effect of retrieval practice—first reported in well-controlled studies more than 100 years ago (e.g., Abbott, 1909a, 1909b)—has remained an important tenet in the scientific literature on memory. In fact, with growing recognition of the potential educational benefits of retrieval practice, the pace at which studies on retrieval have appeared has increased in the past 25 years (see reviews in Adesope et al., 2017; Fazio & Marsh, 2019; Karpicke, 2017; Latimier et al., 2021; Rowland, 2014).

Although the application of retrieval practice is relatively new in the DLD literature, a number of reliable findings have already emerged. For example, consistent with the extant literature with adults and children with TD, children and young adults with DLD learn more words when retrieval practice is included than through passive study alone, even when degree of word exposure is controlled (e.g., Leonard, Karpicke, et al., 2019; Leonard et al., 2023; McGregor, Gordon, et al., 2017). This finding holds whether the words are nouns (Leonard, Karpicke, et al., 2019), adjectives (Leonard, Deevy, et al., 2019), or verbs (Leonard et al., 2023).

In most of these studies, “spaced” retrieval has been employed, defined as a protocol that requires children to retrieve a word after several other words had intervened since the last time the to-be-retrieved word was heard. Along with showing an advantage over passive study alone, spaced retrieval also appears to produce greater word learning gains than a procedure involving immediate retrieval without spacing (Haebig et al., 2019). Spaced retrieval has also shown an advantage over alternative learning procedures, such as rich vocabulary instruction (Levlin et al., 2022).

Some studies have examined word learning both from the standpoint of the children learning the word form itself (e.g., “barracuda” /bærǝkudǝ/) and its meaning (e.g., “a fish with sharp teeth”). This is true as well for studies employing novel word forms (e.g., /bog/) and an arbitrary meaning assigned to each (e.g., “likes rain”). Across studies, word learning gains through spaced retrieval have been greater than gains through comparison procedures for both word forms and meanings, but the magnitude of the difference is usually larger for word forms (e.g., McGregor, Arbisi, & Eden, 2017; see multistudy examination in Leonard et al., 2021). One exception was a study by Adlof et al. (2021) of children 7–9 years of age. These investigators found that children with both DLD and dyslexia showed lower word form accuracy and verbal semantic recall than same-age peers. However, children with DLD without dyslexia scored lower than peers only on verbal semantic recall; their word form accuracy did not differ.

In novel word learning studies, children with DLD often learn fewer novel words than same-age peers with TD, though in some cases, the differences can be statistically accounted for by differences in the children's pre-experiment standardized vocabulary scores. A particularly important finding is that the differences seen between these groups are apparent early in the learning phase but the decline in recall over longer periods is no larger for children with DLD than it is for their peers (e.g., Gordon, Storkel, et al., 2021; Leonard et al., 2021). Group differences from the outset point to encoding problems as perhaps the greatest contributor to these children's word learning weaknesses (Bishop & Hsu, 2015; Gordon, Storkel, et al., 2021; Jackson et al., 2021; McGregor, Gordon, et al., 2017).

When retrieval trials during learning provide the children with partial information about the target word form (e.g., providing the children with the first syllable of the word), higher accuracy is seen on these trials than when the children must retrieve the entire word form with no partial information (Gordon, Storkel, et al., 2021). However, paradoxically, the opposite is true for longer-term retention. When longer-term retention is tested, children show higher scores when, during the learning period, they had to recall the word form with no partial information provided than when they had been given this information (Gordon, McGregor, & Arbisi-Kelm, 2021).

## Retrieval Practice During Shared Book Reading

Much as we have learned about the benefits of retrieval practice for word learning by children with DLD, more needs to be learned to make full use of this promising tool. Thus far, with a few exceptions (e.g., Gordon et al., 2023; Levlin et al., 2022), the studies have been in a laboratory context in which children learn novel words. The tight experimental controls offered by the laboratory setting have enabled many basic findings to be replicated across studies. However, the designs and procedures used in laboratory studies cannot be directly translated into more practical settings without some modifications. In this study, we seek to determine if well-established laboratory findings regarding retrieval practice hold when applied to the context of *shared book reading*. Ours is a relatively small step, as we maintain much of the experimental rigor as in earlier studies but embed the novel words to be learned within stories presented in a children's book.

The advantages of shared book reading are well attested in the child development literature. It is a natural vehicle for caregiver–child or teacher–child interactions. Shared book reading, including dialogic book reading, exposes children to words as well as grammatical details that appear less frequently in day-to-day oral language (Nation et al., 2022; Zevenbergen & Whitehurst, 2003). For example, books used with preschoolers have greater lexical diversity, with longer and more abstract words than appear in the language these children hear in conversation (N. Dawson et al., 2021; Montag et al., 2015). Although studies measuring broad changes in language through shared book reading tend to produce fairly small effect sizes (Noble et al., 2019), studies focusing specifically on word learning often show clear benefits (see meta-analyses in Flack et al., 2018; Marulis & Neuman, 2010; Mol et al., 2008; and a recent study by Rosslund et al., 2024).

Studies of the effects of shared book reading on the word learning of children with DLD have been less frequent in the literature. However, larger-scale studies have recently emerged, including clinical trials (see Storkel et al., 2017, 2019). These studies have shown large individual differences in the number of words children with DLD learn through shared book reading. The factors contributing to this variation are not yet clear, as the measures proving predictive of better word learning are not always the same across studies. For example, Storkel et al. (2017) found that the number of words children learned was correlated with two measures from the Comprehensive Test of Phonological Processing–Second Edition (Wagner et al., 2013) and one subarea of the Diagnostic Evaluation of Language Variation (Seymour et al., 2005). In the later study by Storkel et al. (2019), the number of words learned was correlated with two measures from the Clinical Evaluation of Language Fundamentals–Fourth Edition (Semel et al., 2003). Notably, the measures that showed correlations in the earlier study did not prove to be related in the later study and vice versa. Of course, differences in detail between the two studies might have accounted for these differing results.

The greater diversity and sophistication of the words appearing in children's books can provide an excellent learning opportunity for children (Nation et al., 2022). Yet, the very fact that these words are at a higher developmental level means that they might prove to be more challenging. This would appear to be especially true for children with DLD given these children's well-documented vocabulary limitations. Therefore, the development of procedures that might increase these children's word learning success during shared book reading seems paramount. In this article, we examine whether spaced retrieval is one such procedure.

There are some features of retrieval practice that are quite compatible with shared book reading. As noted by Rosslund et al. (2024), during shared book reading, adults provide more referential language and often ask questions (e.g., Snow et al., 1976; Tamis-LeMonda et al., 2019). Referential language is essential in retrieval practice as children must hear new words and associate these words with particular referents, and the retrieval prompts at the heart of retrieval practice usually take the form of questions. The difference in retrieval practice, of course, is that particular words are targeted and can thus take even more advantage of these seemingly central features of shared book reading.

## Item Spacing With Feedback and Small Set Size

In principle, the application of retrieval practice to shared book reading appears straightforward. However, for children with TD of preschool age, retrieval practice advantages over repeated study (RS) have not clearly emerged in a book reading format (see Knabe et al., 2023). This contrasts with findings from previous (non–book reading) studies, which have shown relative success with retrieval practice for preschool-age children with TD (e.g., Fritz et al., 2007) as well as findings for both typically developing children and children with DLD in our own (non–book reading) laboratory studies. We attribute this relative success to: (a) *item spacing with feedback* and (b) *a small number of words to be learned in each set* during the learning phase. To our knowledge, these features have not been employed together in earlier studies of preschoolers within a shared book reading format. For example, in the study by Knabe et al. (2023), 10 novel words were used, and retrieval was either immediate with no spacing (their Experiment 1), or there was item spacing but a large number of other novel words (nine) appeared between the time a novel word had to be retrieved and the last time it was heard (their Experiment 2).

Findings from both the adult literature and our own work with preschoolers have shaped our use of these two details in our protocol. First, feedback is especially helpful when participants are incorrect or correct but unsure of their immediately preceding retrieval attempt (Butler et al., 2008; Rowland & DeLosh, 2015)—a scenario common with early spaced retrieval items when accuracy is lower. Second, our own findings have shown that learning a set of four words results in more recall success by preschool-age children than a set of six words (compare Leonard, Karpicke, et al., 2019, with Haebig et al., 2019). Third, we have found that spaced retrieval results in better recall than a procedure employing the same number of retrieval opportunities without spacing (Haebig et al., 2019). Fourth, all of the learning conditions that have yielded better word recall than the comparison conditions in our work have employed item spacing during the learning phase, in which a new word had to be retrieved after two to three other words had intervened since the last time the to-be-retrieved word was heard.

**Figure 1.**
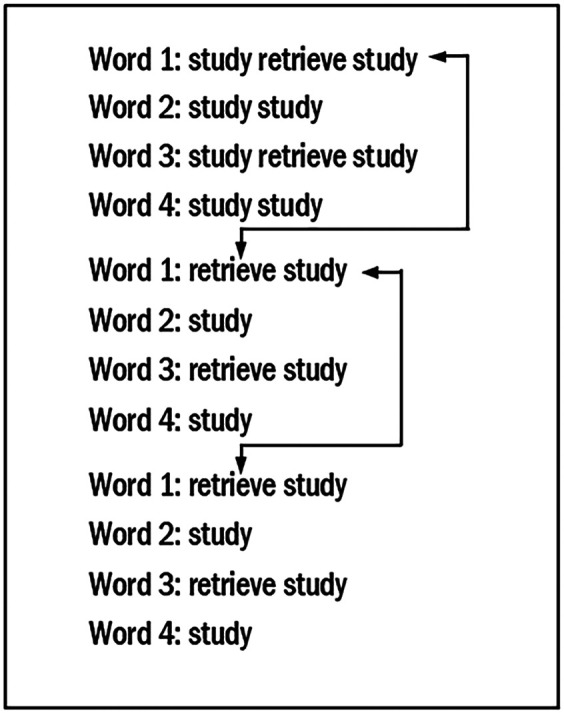
Protocol for the first block on the first day in the work of Leonard, Karpicke, et al. (2019). Black arrows = retrieval with item spacing.

We illustrate these features in Figure 1, which reflects the design used in Leonard, Karpicke, et al. (2019). In that study, 4- and 5-year-old children with DLD and their same-age peers learned each set of four novel words over two sessions held on consecutive days. Two of the novel words appeared in a spaced retrieval condition and two in an RS condition with no retrieval opportunities. The example in Figure 1 shows the sequence for the first block on the first day. For the spaced retrieval condition, the first retrieval trial involved immediate retrieval; the child heard the word while seeing its corresponding referent (a study trial), and then the referent was immediately presented again, and the child was asked to recall the novel word (a retrieval trial). This was followed by a study trial for the same word, which also served to provide feedback. This is indicated as “study–retrieve–study” in Figure 1. For subsequent retrieval trials of the same word, spacing was used; three other novel words were heard between the time the to-be-retrieved word was last heard and when it was to be retrieved. This is shown by the black arrows. To simplify the illustration, we use arrows only for Word 1, but the spacing applies as well to Word 3, the other word in the spaced retrieval condition. Each of these spaced retrieval trials was followed by a study trial. In contrast, words assigned to the RS condition (Words 2 and 4) have no retrieval trials but have the same number of study trials. In the Leonard, Karpicke et al. study, the sequence in Figure 1 is repeated a second time on the first day and two more times on the second day. Five minutes after the session on the second day, a recall test is administered.

**Figure 2.**
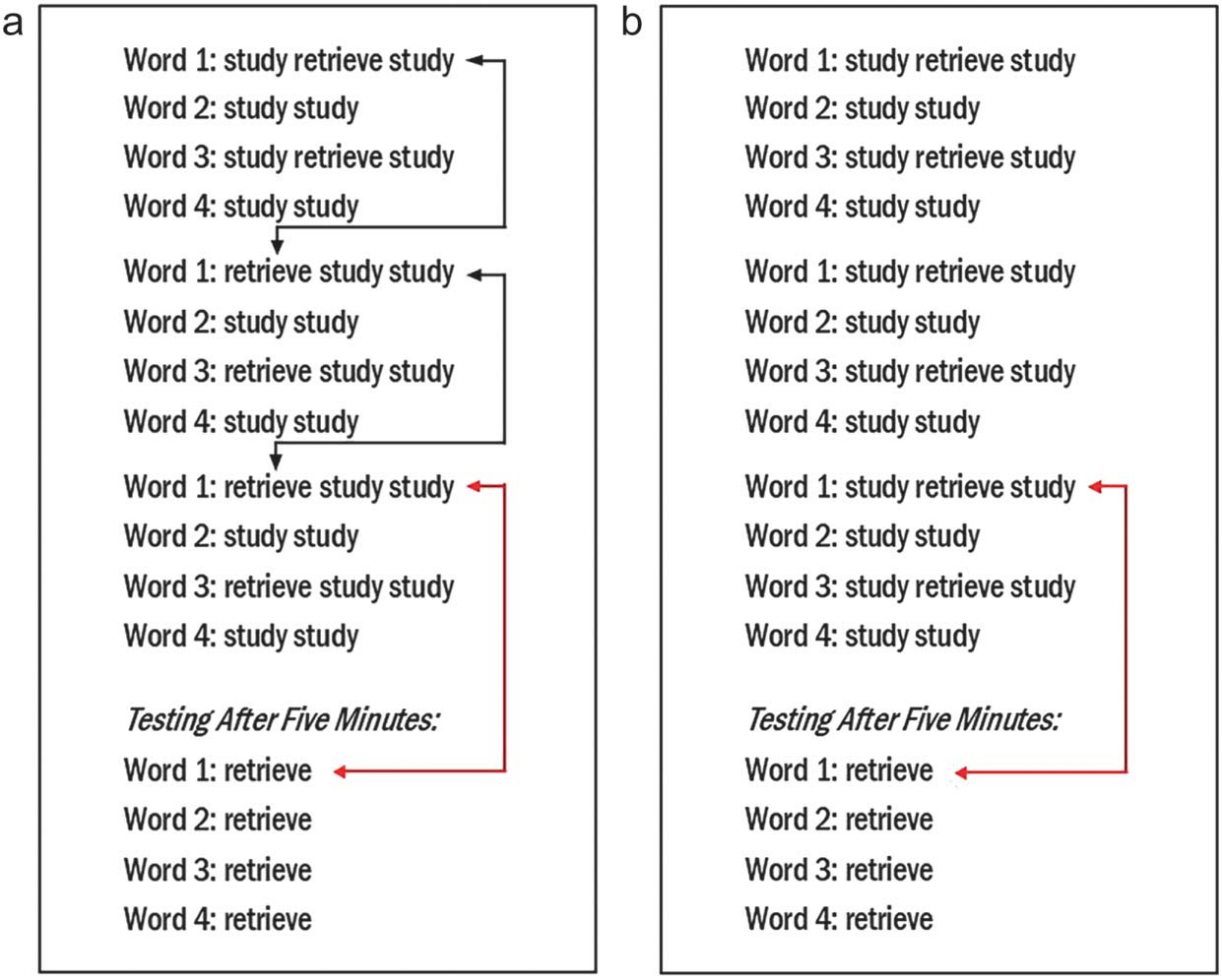
Panel a: Spaced retrieval protocol with item spacing during the learning period. Black arrows = retrieval with item spacing; red arrows = retrieval with item spacing plus intervening delay. Panel b: Protocol providing no early experience with retrieval with item spacing. Red arrow = retrieval with item spacing plus intervening delay.

The recall test necessarily involves two kinds of spacing—the spacing due to the 5-min break and the spacing due to the intervening words since the last time the to-be-retrieved word was heard. We illustrate this point in Figures 2a and 2b by contrasting two hypothetical protocols that are matched for both the number of retrieval opportunities and the number of study trials during the learning period. Note that in each protocol, there is a study trial that immediately follows each retrieval trial, thus equating the two protocols also on the amount of feedback provided. This arrangement makes the two protocols different in only one key respect—whether retrieval during the learning period includes spacing.

Spaced retrieval is used during the learning period in the protocol shown in Figure 2, as indicated by the black arrows. The red arrow in Figure 2 indicates that when testing occurs after 5 min, the children must still contend with the fact that three other novel words had intervened since the last time the to-be-retrieved word was heard. However, this challenge might be partially reduced with the practice with spaced retrieval provided during the learning period—practice that should strengthen the recall of these words before recall testing begins. The protocol shown in Figure 2b provides no such benefit. Here, all retrieval trials involve immediate retrieval. Without prior practice with spacing (note the absence of black arrows), when tested, the children must retrieve novel words after both a time delay and three other novel words intervening since the last time the to-be-retrieved word was heard.

We believe that by including spacing during the learning period in our studies, along with feedback and a small word set size, we have been able to see gains in the spaced retrieval condition that exceeded those seen in comparison conditions. Accordingly, we follow the same scheme in the present study, though adapted to a shared book reading format.

## Are Newly Learned Word Forms Morphologically Flexible?

In previous studies with novel verbs, children with DLD were significantly limited in their ability to add inflections to novel verbs learned in bare stem form (e.g., adding *–ing* to *nepp*) or, conversely, converting words learned with inflections to their bare stem form (*nepping* to *nepp*) when the sentence context required it (Leonard et al., 2023, 2024). Same-age peers with TD had no difficulty making these changes. These studies have been confined to verb learning—a notoriously difficult area for children with DLD. In this study, we had the opportunity to observe whether this apparent morphological inflexibility applied as well when novel words took the form of nouns. After the children were tested on the recall of the novel words in their originally presented form (e.g., /dɔm/), we assessed the children's ability to produce the plural form of the novel verb (e.g., /dɔmz/), when presented with multiple exemplars of the novel word's referents.

## Research Questions

In this study, we ask if retrieval practice can be successful when applied to a shared book reading context. Our main questions are:

1. Will 4- and 5-year-old children with DLD, like their peers with TD, show greater learning of novel nouns when spaced retrieval is employed than when the novel nouns are studied without retrieval?
2. Will this superior learning be seen across the different measures of word form recall, meaning recall, and recognition?
3. Will this superior learning hold both when testing occurs directly after learning and 1 week later?
4. As an additional question, we ask, “Will children with DLD be successful when the correctly recalled (singular) novel nouns must be changed to their plural form?”  

## Method

### Overview

This study was registered at clinicaltrials.gov (Laurence B. Leonard, NCT06026124). The recruitment and experimental procedures used here were approved by the Purdue University Institutional Review Board (IRB# 1603017480). Written consent was obtained from the children's families, and verbal assent was provided by the children.

Though presented in a shared book reading context, most of the design features of the study were based on Leonard, Karpicke, et al. (2019). The same types of novel words, referents, word exposures, retrieval prompts, item spacing, and feedback were used. Two sets of novel words were used, and within each set, two novel words were assigned to a spaced retrieval condition, and two were assigned to an RS condition. Each set of four words appeared in a different story. The words and referents in each condition appeared in the story as exotic plants and unusual animals seen along the way during the main story characters' journey, rather than playing a role in the story line itself. When the character happened upon the referent, the child heard both the word form and what it likes (e.g., “This is a /gɪs/. It's a /gɪs/. A /gɪs/ likes trees”). This constituted a study trial. The story then continued with no further mention of the referent until the referent was encountered again. When retrieval trials were called for, the referent appeared and the child was asked, “What's this called? What do we call this?” for word form, and, after the child's response, “What does this one like? What does it like?” for “meaning.” The story used for each set was presented twice on the first day and twice on the second day. Recall testing occurred 5 min after the second reading of the story on the second day. During the 5-min interval, the child played with one of several toys or games introduced by the experimenter. One week later, the recall tests were re-administered. Also at the 1-week mark, we tested the children's ability to inflect each novel word with noun plural –*s*, as in, “Here is one dog and here are three … (/paɪbz/).” Finally, we assessed the children's ability to recognize the novel word (“Which one is the /bog/?”) when presented with an array of drawings of three of the referents.

### Participants

Twenty-seven children served as participants. Thirteen met the selection criteria for DLD, and 14 met the criteria for TD. The children in the two groups were similar in age (DLD: *M* = 59.13 months, *SD* = 7.57; TD: *M* = 57.07 months, *SD* = 6.76). All children in both groups scored above 75 on the Primary Nonverbal Test of Intelligence (Ehrler & McGhee, 2008) and passed a hearing screening in both ears at 20 dB at 500, 1000, 2000, and 4000 Hz.

The children in the DLD group (six girls, seven boys) were already enrolled in a language intervention program or scheduled to begin such a program. Each child in this group scored below 87 on the Structured Photographic Expressive Language Test–Preschool 2 (SPELT-P2; J. Dawson et al., 2005)—the empirically derived cutoff score reflecting good sensitivity and specificity (Greenslade et al., 2009). All children in the group scored in the “minimal-to-no-symptoms of autism spectrum disorder” range on the Childhood Autism Rating Scale–Second Edition (CARS-2; Schopler et al., 2010).

Six girls and eight boys comprised the TD group. All scored above 87 on the SPELT-P2. No problems with development in general or with language in particular were reported for these children. Given no concerns about these children's developmental histories, the CARS-2 was not administered to this group.

In addition to the selection criterion measures noted above, we administered three other measures for descriptive purposes, two of which served as covariates, as in our previous work. One covariate was the standard score on the Peabody Picture Vocabulary Test–Fourth Edition (PPVT-4; Dunn & Dunn, 2007); the other was maternal education measured in years. In some of the earlier studies (e.g., Leonard et al., 2023), the covariates reduced the group (DLD vs. TD) effect, whereas in others (e.g., Leonard et al., 2024), they had no influence on the results. Table 1 provides a summary of the children's scores on the selection criterion and descriptive measures.

**Table 1.** Summary of the test scores and related information obtained from the children with developmental language disorder (DLD) and with typical language development (TD).

Variable	DLD (*n* = 13)	TD (*n* = 14)
Age in months	59.13 (7.57)	57.07 (6.76)
Sex	6 F, 7 M	6 F, 8 M
SPELT-P2 (SS)^a^	67.15 (12.99)	120.50 (6.55)
PTONI (SS)^a^	107.38 (14.36)	120.93 (12.31)
PPVT-4 (SS)^b^	100.00 (10.20)	121.57 (10.60)^c^
Maternal education in years^b^	17.23 (3.77)	18.04 (2.31)

*Note.* F = female; M = male; SPELT-P2 = Structured Photographic Expressive Language Test–Preschool 2; SS = standard score; PTONI = Primary Test of Nonverbal Intelligence; PPVT-4 = Peabody Picture Vocabulary Test–Fourth Edition; PPVT-5 = Peabody Picture Vocabulary Test–Fifth Edition.

a Selection criterion measure.

b Covariate measure.

c Two children in this group received the PPVT-5.

Finally, to assist our scoring of the children's production of the novel words, we administered a customized real-word production task that emphasized the same initial and final consonants used for the novel words. The task involved the children repeating short phrases in which the relevant real word appeared in final position (e.g., “Say ‘fly a kite’”). When taken together, the real words reflected the same phonemes in the same word positions as the novel words. For example, for the novel word /jʌt/, the real words *you* and *kite* were included. Our scoring procedure (see below) took common developmental errors into account, but the production task allowed us to identify any unusual production patterns that might make scoring less straightforward.

### Novel Words and Object Referents

Eight novel words were used, divided into two sets of four. In each set, two novel words were assigned to the spaced retrieval condition and two to the RS condition. Through counterbalancing, each novel word appeared in both conditions across children in each group. The novel words were the consonant–vowel–consonant monosyllables: /paɪb/, /nɛp/, /faʊn/, /jʌt/, /wæd/, /bog/, /dɔm/, and /gɪs/. The first six of these were also used in the laboratory study on which the current study was based (Leonard, Karpicke, et al., 2019). No two novel words shared the same initial consonant, vowel, or final consonant. The novel words in the two conditions were matched according to average biphone frequency and neighborhood density based on Storkel (2013). The referents for the novel words were drawings of exotic plants and rare animals. These referents were drawn to closely resemble the photographs of the same plants and animals used in Leonard, Karpicke, et al. (2019).

The number of study trials and retrieval trials matched that used in Leonard, Karpicke, et al. (2019), as did the wording of these trials. For both the spaced retrieval and RS conditions, there were 16 study trials for each novel word. The study trials used the wording, as in “This is a /bog/. It's a /bog/. A /bog/ likes rain.” As can be seen, for each study trial, there were three exposures of the word form (e.g., /bog/) and one exposure to the “meaning” (defined as what the referent liked, as in rain), resulting in a total of 48 exposures of the word form and 16 exposures of the meaning for each word in each condition. For the spaced retrieval condition only, there were 12 retrieval trials. These trials used the wording, “What's this called? What do we call this?”

### Spacing

The pattern of spacing followed the scheme used in Leonard, Karpicke, et al. (2019). The novel words in the two conditions appeared in alternating order, with counterbalancing of the conditions assigned to each word. The two sets of four novel words were learned in sequence with 1 week separating the completion of testing for one set and the introduction of the second set. The learning period for each set was divided into four blocks, with two blocks presented the first day followed by the remaining two blocks on the next day. A 5-min break was provided between the two blocks of the same day.

Within each block, and as illustrated in Figure 1, the first retrieval trial for each novel word in the spaced retrieval condition was an immediate retrieval trial. The children were presented with a study trial, and this was followed directly by a retrieval trial. Another study trial for the same novel word followed, which provided feedback. This study trial appeared regardless of the accuracy of the child's retrieval attempt. The remaining two retrieval trials for the novel word in each block were spaced trials. For these trials, three other novel words had been presented since the last time the word-to-be-retrieved had appeared in a study trial. Within each block, then, the spacing for each novel word in the spaced retrieval condition can be referred to as “0–3–3” with the number referring to the number of intervening words.

For the novel words in the RS condition, the first study trial for each novel word in each block was directly followed by another study trial for the same novel word. For the remainder of the block, only one study trial was provided for each novel word. This arrangement allowed the child to hear the novel words in the RS condition the same number of times as they heard the novel words in the spaced retrieval condition.

### Stories

Two stories were used, one for each set. Each story was read 4 times, 2 times on the first day and 2 times on the second day. A 5-min break was given between each reading on the same day. The stories were presented on a laptop computer in a testing room designed for interacting with children. Each page of the story was “turned” by the experimenter using a computer key. Each page showed an artist's drawing of the events in the stories as well as the exotic plants and rare animals serving as referents for the novel words. For each story, there was a title page and 33 pages constituting the story.

One story was an adaptation of *My Dinosaur* by Mark Alan Weatherby (1997). In this story, when there is a full moon, a young girl waits for her friend, a dinosaur, to pick her up. She then rides the dinosaur through the forest. As they move through the forest, they see the exotic plants and rare animals serving as the referents. Eventually, as the sun starts to come up, the dinosaur brings the little girl back to her house. During each reading of the story, there were 12 exposures of each novel word form and four exposures of the meaning (what the plant or animal likes) distributed in four study trials. With four readings of the story, this amounted to 48 exposures of the word forms and 16 exposures of the meanings during study trials (for each word regardless of condition). For each novel word in the spaced retrieval condition, there were three retrieval prompts during each reading, with a total of 12 retrieval prompts across the 2 days. During each reading, the first retrieval trial was an immediate (“0”) retrieval trial; the subsequent retrieval trials involved “3” spacing.

The second story was an adaptation of *Sam and Dave Dig a Hole* by Mac Barnett (2014). The two boys, Sam and Dave, decide to dig a hole and continue to dig until they find something “amazing.” They proceed to dig straight down, then sideways, and in the process come across the exotic plants and unusual animals serving as the novel words' referents. At one point, they take a break and have cookies and milk. Further into their digging, they become tired and fall asleep. Just then, the ground gives way and they fall, not deeper underground but (surprisingly) on to their own backyard. They then go into the house for chocolate milk and cookies. The number and distribution of the study and retrieval trials were the same as in the first story.

## Postlearning Tests

Five minutes after the second block on the second day, the children's recall of the novel word forms and meanings was tested. Each novel word form and meaning was tested twice, (with the second item for each word form and meaning occurring only after all four novel word forms and meanings had been tested once). Recall testing used the same prompts as used on retrieval trials (“What's this called? What do we call this?” and “What does this one like? What does it like?”). One week later, the test for word form and meaning was repeated. In addition, the children with DLD (only) were assessed on their ability to both recall the correct novel word and inflect it with the plural –*s* inflection when shown a drawing of multiple exemplars of the referent. The task was in the form of a simple sentence completion task. The examiner described a picture of a single familiar object and began to describe a picture with three novel objects. The child had to complete the sentence by providing the plural form of the appropriate novel word (e.g., Examiner: “Here is one shoe and here are three … ” Child: “/dɔmz/”). There were 18 items on the plural task. Each of the four novel words was assessed with three items. The six remaining items required the children to provide the plural form of familiar nouns (e.g., *cars*, *shoes*). Finally, also at 1 week, a recognition test was administered. Eight items were used. For each item, the drawing of the correct referent appeared on the screen along with drawings of two other referents from the same set. The child was then asked to point to the correct drawing upon hearing the prompt, for example, “Which one is the /gɪs/? Where's the /gɪs/?”

### Scoring and Reliability

Scores on the 5-min and 1-week recall tests for word form were the number of items judged as correct. However, as in the earlier work, we did not require error-free pronunciation of the novel word to deem a production “correct.” Scoring of each production followed three steps. First, the production could not be interpreted as a real word that could serve as a reasonable label for the referent (e.g., “flower”). Second, based on subjective judgment, the production had to seem like a plausible attempt at the novel word. At this point, we applied a scoring system based on Edwards et al. (2004). In this system, the production is judged against adult pronunciation. Consonants are awarded 1 point each for matching the adult form in place, manner, and voicing. Vowels are given 1 point each for matching the adult form according to backness, height, and length. An additional point is credited if the production has the same syllable shape as the adult form, which for all novel words was consonant–vowel–consonant. The total score for each production is then compared to a score that would be assigned if the production were instead an attempt at one of the other novel words. For example, the pronunciation of the novel word /faʊn/ as /faʊm/ would earn a score of 3 + 3 + 2 + 1 = 9, with 1 point deducted for the error in place of articulation for the final consonant. The pronunciation /faʊp/ would receive a score of 7 (3 + 3 + 0 + 1) with 3 points deducted due to the place, manner, and voicing errors in the final consonant. If /faʊm/ was assumed to be an attempt at the novel word /dɔm/, for example, only 6 points would be awarded (0 + 2 + 3 + 1 = 6) based on the errors in the initial consonant and vowel. If lower scores than /faʊm/ were also seen for other comparisons, /faʊm/ would be treated as a correct response. Instances were rare in which a subjectively judged attempt at the target novel word had no higher score than if the production was assumed to be alternative novel word. However, this method served as a safeguard against overly liberal subjective judgments in favor of the target novel word.

These scoring criteria produce reliable interjudge agreement (see below) and hold advantages over other criteria. For example, a criterion of 100% phonetic accuracy would treat a production such as /faʊm/ for /faʊn/ as incorrect and not distinguishable from the child's production of the wrong novel word. An alternative such as the use of a 0%–100% continuum (e.g., percentage phonemes correct or percentage features correct) would provide no qualitative distinction between an imprecise production of the correct novel word and a production that cannot be reliably judged as an attempt at the correct novel word. A score of, say, 60% correct by itself is no assurance that the child was actually attempting to produce the correct novel word. We did not require similar criteria for scoring accuracy on the meaning recall tests. Responses such as “sun,” “trees,” and “birds” were easily distinguishable. Adult pronunciation was not required.

To assess interjudge scoring reliability of the children's word form recall at 5 min and 1 week, an independent judge scored the responses of four children with DLD and four children from the TD group. With two sets, each with a 5-min and 1-week test and eight items on each test, there were 32 items per child and 256 items across the eight children. Agreement on correct/incorrect judgments was 99%.

For the plural task, administered only to the children with DLD, an independent judge scored the responses of four children. On this task, there were 12 items in each set. With 24 items for each of the four children, there was a total of 96 items. The items were scored in two ways. First, the number of items on which the child recalled the correct novel word was determined. Second, of that number, the number that was correctly produced with a plural form was noted. Agreement on both the recall of the correct novel word and the number produced with a plural form was 100%.

### Data Analysis

To assess the children's recall and recognition of the novel words, a series of mixed-effects models were estimated with a random intercept at the child level, with repeated measures nested within a child. We included random slopes for learning condition and time when they did not approximate zero. For the measures of word form recall and meaning recall, the model included participant group (DLD, TD), learning condition (repeated spaced retrieval [RSR], RS), and time (5 min, 1 week). For the recognition measure, only participant group and learning condition were included, as recognition was assessed only at 1 week. For each measure, we present models with and without the covariates of PPVT-4 standard scores and maternal education. Also, for each measure, we tested main effects models and additional models that included two- and three-way interactions with the covariates. To correct for deviations from normality for each measure, bootstrapped standard errors with 1,000 replicates were estimated. In the Results section, we present only those models that proved informative, and in describing details of the data, we focus on the models with covariates. Partially standardized regression coefficients (*b*_std_) are provided as effect sizes. These are comparable to Cohen's *d* but are conditioned on the model covariates. Best models were chosen based on inclusion of interactions when they were statistically significant at *p* < .05. Stata/SE (Version 18.0) was used for estimating models (StataCorp, 2023).

The plural –*s* task was administered only to the children with DLD and was viewed as a supplementary task—an opportunity to assess whether the children could add an inflection to the novel words they had learned. We compared the novel words in the two conditions according to the degree to which the novel words that were recalled were inflected with plural –*s*.

## Results

### Word Form Recall

**Figure 3.**
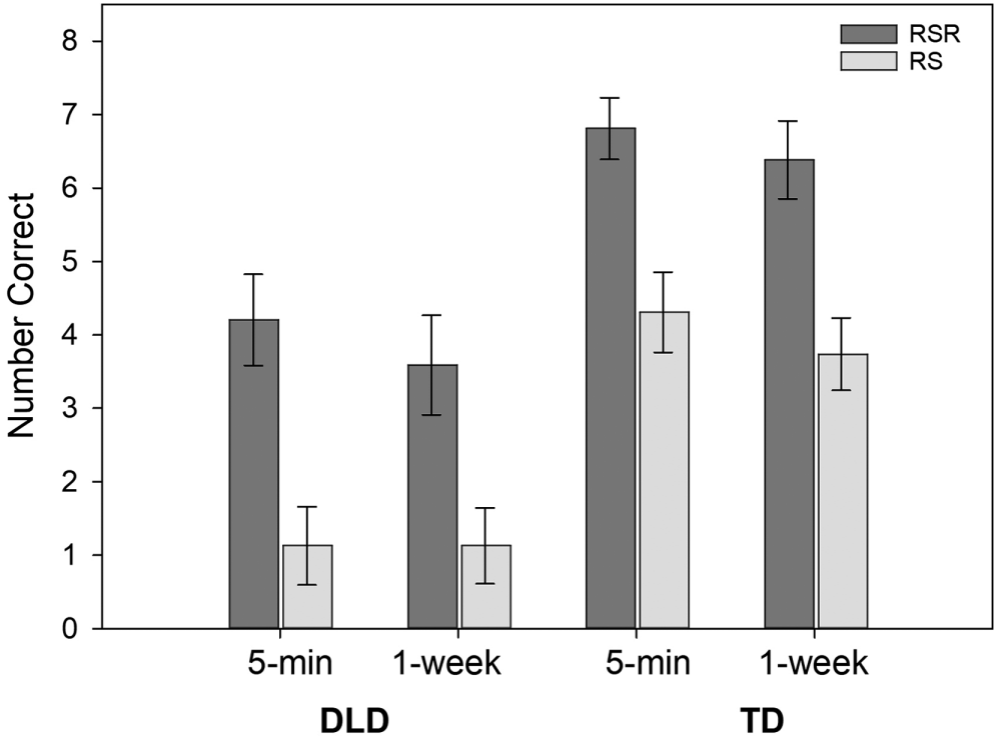
The mean number of word form items correct on the recall test at 5 min and 1 week for novel words in the repeated spaced retrieval (RSR) condition and the repeated study (RS) condition. Error bars are standard errors. DLD = children with developmental language disorder; TD = children with typical language development.

For word form recall, the best model was the main effects model. Two- and three-way interactions were not close to statistical significance. Model estimates appear in Table 2. The effect size for learning condition was very large (*b*_std_ = 0.98), with recall of novel words in the RSR condition considerably better than recall of novel words in the RS condition by approximately 2.67 points (out of 8). The group effect (favoring the children with TD by approximately 2.85 points) was likewise large (*b*_std_ = −1.05). A small effect size was also seen for time (*b*_std_ = −0.15) and reflected a small decline in recall from 5-min to 1-week testing (a difference of approximately 0.41 points). There was significant between-child variance in the condition effect as estimated by the random slope for condition, indicating that some children showed larger differences between their RSR and RS recall than other children. An illustration of the data appears in Figure 3.

**Table 2.** Main effects word form model results (*N* = 27, *o* = 108).^a^

Variable	Main effects: no covariates					Main effects: with covariates				
*Fixed effects*	*b*	95% CI		*b*_std_	*p* value	*b*	95% CI		*b*_std_	*p* value
Group (DLD vs. TD)	−2.23	−3.31	−1.14	−0.82	.000	−2.85	−4.54	−1.17	−1.05	.001
Condition (RSR vs. RS)	2.67	2.16	3.17	0.98	.000	2.67	2.18	3.16	0.98	.000
Time (1 week vs. 5 min)	−0.41	−0.70	−0.11	−0.15	.007	−0.41	−0.72	−0.09	−0.15	.011
Covariates										
PPVT						−0.02	−0.08	0.03	−0.01	.356
Mother's education						−0.07	−0.28	0.14	−0.03	.506
Intercept	3.91	3.15	4.66		.000	8.16	1.21	15.11		.021
***Random effects***	**σ** ^**2**^	**95% CI**				**σ** ^**2**^	**95% CI**			
Condition	2.19	1.31	3.63			2.20	1.32	3.67		
Intercept	2.95	1.54	5.65			3.11	1.57	6.19		
Residual	0.79	0.46	1.36			0.79	0.45	1.38		

*Note.* CI = confidence interval; DLD = children with developmental language disorder; TD = children with typical language development; RSR = repeated spaced retrieval; RS = repeated study; PPVT = Peabody Picture Vocabulary Test.

a Bootstrap standard errors with 1,000 replicates.

### Meaning Recall

**Figure 4.**
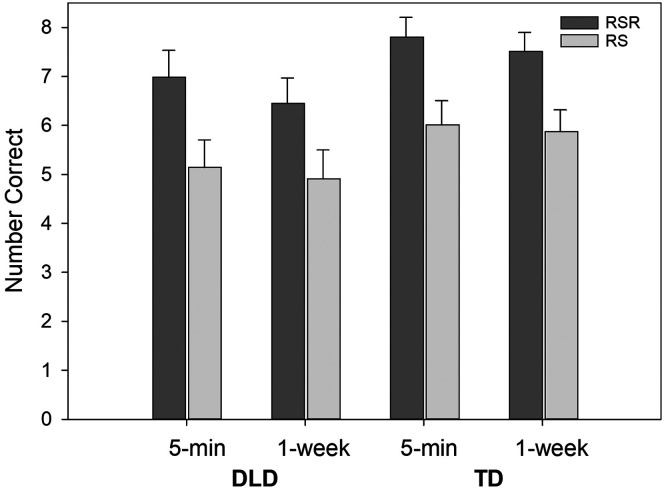
The mean number of meaning items correct on the recall test at 5 min and 1 week for novel words in the repeated spaced retrieval (RSR) condition and the repeated study (RS) condition. Error bars are standard errors. DLD = children with developmental language disorder; TD = children with typical language development.

The best model for meaning recall was the main effects model, shown in Table 3. The two- and three-way interactions were not significant. The learning condition effect reached statistical significance and showed a large effect size (*b*_std_ = 0.91). Recall scores for the RSR condition were about 1.70 points higher than recall scores for the RS condition. The group difference became larger with the model that included the covariates but did not reach the .05 α level. As can be seen in Table 3, the time effect did not reach the .05 level and showed only a small effect size. There was significant between-child variance in both the learning condition effect and the time effect. Figure 4 illustrates the data.

**Table 3.** Main effects meaning model results (*N* = 27, *o* = 108).^a^

Variable	Main effects: no covariates					Main effects: with covariates				
*Fixed effects*	*b*	95% CI		*b_std_*	*p* value	*b*	95% CI		*b_std_*	*p* value
Group (DLD vs. TD)	−0.28	−1.33	0.77	−0.15	.605	−0.78	−2.52	0.95	−0.42	.376
Condition (RSR vs. RS)	1.70	1.26	2.15	0.91	.000	1.70	1.24	2.17	0.91	.000
Time (1 week vs. 5 min)	−0.30	−0.61	0.01	−0.16	.062	−0.30	−0.61	0.01	−0.16	.061
Covariates										
PPVT						−0.01	−0.07	0.05	−0.01	.659
Mother's education						−0.16	−0.30	−0.03	−0.09	.017
Intercept	5.78	4.92	6.64		.000	10.32	3.05	17.59		.005
***Random effects***	**σ** ^**2**^	**95% CI**				**σ** ^**2**^	**95% CI**			
Condition	1.39	0.95	2.03			1.40	0.95	2.07		
Time	0.28	0.16	0.50			0.24	0.13	0.42		
Intercept	2.22	1.48	3.35			2.11	1.26	3.53		
Residual	0.74	0.37	1.47			0.75	0.39	1.46		

*Note.* CI = confidence interval; DLD = children with developmental language disorder; TD = children with typical language development; RSR = repeated spaced retrieval; RS = repeated study; PPVT = Peabody Picture Vocabulary Test.

a Bootstrap standard errors with 1,000 replicates.

### Recognition

**Figure 5.**
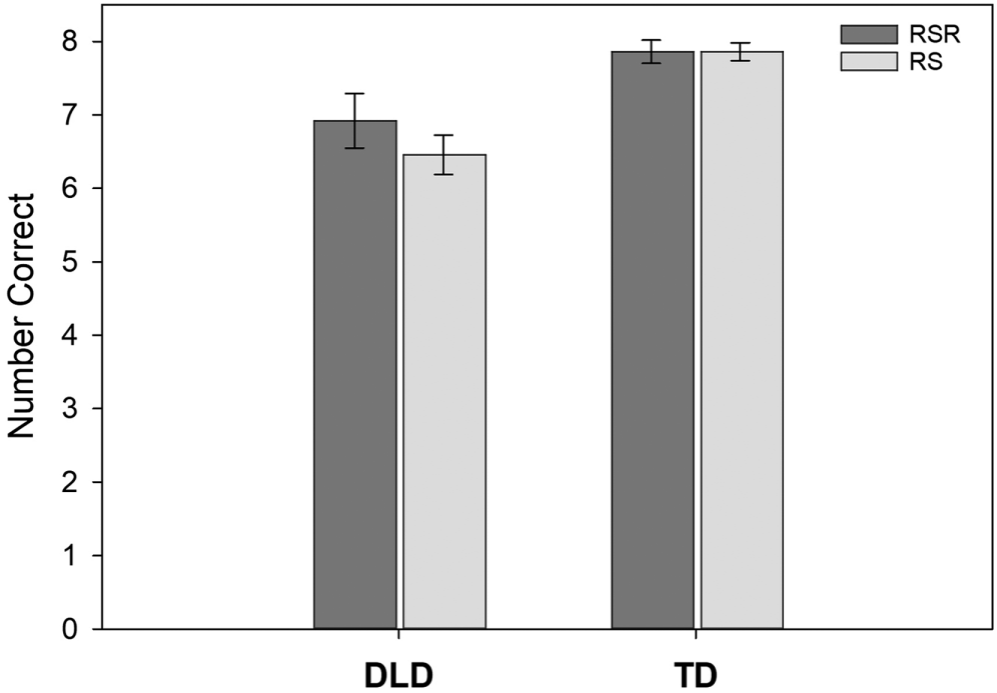
The mean number of items correctly identified on the recognition test administered at the 1-week point for novel words in the repeated spaced retrieval (RSR) condition and the repeated study (RS) condition. Error bars are standard errors. DLD = children with developmental language disorder; TD = children with typical language development.

Recognition was assessed only at the 1-week point. Again, the best model was the main effects model (see Table 4). The two-way interaction (Group × Learning Condition) was not significant. In contrast to the previous analyses, no learning condition difference appeared, only a significant group effect (*b*_std_ = −1.04). Recall scores for the TD group were approximately 1.22 points higher than recall scores for the DLD group. There was significant between-child variance in the learning condition effect. Figure 5 provides an illustration of the data.

**Table 4.** Main effects recognition model results (*N* = 27, *o* = 54).^a^

Variable	Main effects: no covariates					Main effects: with covariates				
*Fixed effects*	*b*	95% CI		*b_std_*	*p* value	*b*	95% CI		*b_std_*	*p* value
Group (DLD vs. TD)	−1.24	−1.79	−0.69	−1.06	.000	−1.22	−1.83	−0.62	−1.04	.000
Condition (RSR vs. RS)	0.22	−0.12	0.56	0.19	.200	0.22	−0.11	0.55	0.19	.187
Covariates										
PPVT						0.00	−0.02	0.03	0.00	.910
Mother's education						−0.01	−0.15	0.13	−0.01	.919
Intercept	7.78	7.64	7.93		.000	7.73	3.81	11.65		.000
***Random effects***	**σ** ^**2**^	**95% CI**				**σ** ^**2**^	**95% CI**			
Condition	0.47	0.23	0.94			0.48	0.23	1.00		
Intercept	0.37	0.17	0.79			0.43	0.15	1.22		
Residual	0.47	0.00	447.59			0.47	0.00	92.02		

*Note.* CI = confidence interval; DLD = children with developmental language disorder; TD = children with typical language development; RSR = repeated spaced retrieval; RS = repeated study; PPVT = Peabody Picture Vocabulary Test.

a Bootstrap standard errors with 1,000 replicates.

### Plural –*s* Task

The plural –*s* task, like the recognition test, was administered at the 1-week mark. However, only the children with DLD were administered this task. More of the novel words from the RSR condition (*M* = 4.85, *SD* = 3.93) were inflected with plural –*s* than novel words from the RS condition (*M* = 2.08, *SD* = 2.75), *t*(12) = 2.803, *p* = .016. However, this is not surprising, given that more of the RSR novel words overall (*M* = 5.46, *SD* = 4.67) were recalled than RS novel words (*M* = 2.15, *SD* = 2.76), *t*(12) = 3.107, *p* = .009. The more informative observation is that the tendency to inflect the recalled novel words was similarly high in the two conditions—89% and 96% for the RSR and RS words, respectively.

## Discussion

In this study, we asked if retrieval practice could facilitate the novel word learning of children with DLD and their peers when applied to a shared book reading context and, if so, whether this benefit would be seen across different measures of learning (word form recall, meaning recall, recognition) and time (5 min, 1 week). An additional question concerned the children with DLD in particular: Could these children change the correctly recalled novel nouns to their plural forms when the context required it?

### Learning Condition Differences

For both word form and meaning, a clear advantage for RSR was seen. This advantage was seen not only on the 5-min test but also 1 week later. We did not observe a condition effect for the recognition test. In previous studies, recognition tests tend to show the smallest effect sizes favoring RSR relative to word form and meaning recall (see multistudy examination by Leonard et al., 2021), and in a few studies, no condition difference was seen at all for recognition (e.g., Leonard et al., 2023). However, in this as in the other studies, we cannot rule out the possibility that ceiling effects prevented any potential learning condition differences that could have occurred in the TD group in particular. Eleven of the 14 TD children had 100% accuracy for both conditions.

The results from the word form and meaning recall tests suggest that application to a shared book reading activity can preserve the spaced retrieval advantage, at least when the same types and number of novel words, referents, word exposures, retrieval prompts, item spacing, and feedback are used. Although this was our hypothesis, it was certainly possible that the very appearance of the exotic plants and animals within a story could have made their names and meanings sufficiently memorable to result in no additional benefit from spaced retrieval.

The RSR advantage was relative to an RS condition. We believe RS was a useful comparison because children listening to a story with new words while looking at a book is a common activity. In fact, the importance of children hearing new words is often emphasized in the child development literature (e.g., Nation et al., 2022). The advantage shown by RSR, then, suggests that incorporating spaced retrieval in an already recommended practice might provide additional benefits to the children.

It is possible that the magnitude of the difference between the two conditions could have been greater if we had used a different design. We chose to have the RSR words and the RS words appear in alternating order within the same book reading. This means that after being asked to retrieve one of the RSR words and then seeing the picture of the next referent, children might have anticipated having to retrieve that next word. The retrieval prompt did not come because the word was in the RS condition. However, the appearance of the referent might have prompted at least covert rehearsal and made our RS condition a “study-only” condition only in the overt sense.

As in our earlier studies, the children's recall of meanings was clearly superior to that of word forms, yet, meaning recall, like word form recall, benefited from RSR. The finding of stronger meaning recall is well documented in the DLD literature, across the age span (e.g., Leonard et al., 2020; McGregor, Arbisi, & Eden, 2017). At the same time, we acknowledge that our meaning recall test was only a simple association task—one of associating a verbally provided attribute of liking a particular thing (snow, butterflies, etc.) with the picture of the referent, not the name of the referent. This might have provided an exaggerated impression of the children's ability to recall meanings associated with new words.

Even when, as in other studies, the meaning recall advantage is seen with more rigorous semantic tasks, we should be mindful that meaning and word form encoding may not be independent processes. For example, Alt and Plante (2006) found that children with DLD, unlike their same-age peers, recalled fewer semantic features of words (color, shape, pattern, with/without eyes) when the word forms were constructed of phonotactically infrequent sequences. Evidently, the more challenging word forms interfered with a more complete semantic encoding of the words on the part of the children with DLD.

One clinical implication of this word form–meaning learning difference is that we need to be certain that our criterion for concluding that a child has learned a word is the ability to recall the meaning *and* its form, not just the meaning. Inspection of data from both groups showed instances in which the meaning of a word was retrieved rather consistently, but the form was never retrieved correctly during the learning period or during the subsequent recall tests.

As noted earlier, in previous studies of preschoolers with TD, retrieval practice in stories has not shown a clear advantage (e.g., Knabe et al., 2023). Earlier, we noted that our modification of using item spacing during learning with a small set of words was likely to produce more promising results for retrieval practice within a shared reading context. This seemed true, though we did not put our assumptions to a direct test in this study. Instead, we relied on earlier studies from our lab that showed that: (a) learning four words in a set produced greater recall than learning six words in a set, even though spacing was used in both instances (compare Leonard, Karpicke, et al., 2019 with Haebig et al., 2019), and (b) learning words with spaced retrieval during the learning period produced better recall than immediate retrieval with no spacing (compare the two conditions in Haebig et al., 2019). Although in the present study the number of words to be learned was the same in the two conditions, the words in the RSR condition held an advantage because the children received practice retrieving these words with spacing during the learning period. Such practice could have strengthened the children's recall of these words, putting the words in a better position to be recalled during the recall test. In contrast, the children had no such experience with words in the RS condition yet had to retrieve these words with spacing during the recall test 5 min later.

### Recall Over Time

For word form, in particular, there was a small but statistically reliable decline in recall from the 5-min to the 1-week test. This was a main effect, applying to both groups of children with no indication that the decline was larger in the DLD group. Forgetting seemed to be no more a part of the DLD response pattern than it was for the TD pattern—a finding that echoes the results of previous studies (e.g., Gordon, Storkel, et al., 2021; Leonard et al., 2021). Certainly this interpretation applies to the recall of meaning as well, given that neither group showed a decline in meaning recall over the 1-week period.

A common finding in the adult memory literature is that the benefits of spaced retrieval are more likely to be evident in longer-term recall than in recall assessed shortly after the learning phase (Karpicke & Roediger, 2007; Latimier et al., 2021). In the present study, we found advantages for RSR on the 5-min test as well as on the test 1 week later. However, this does not run counter to the prevailing findings from studies on adults. Our first test of the children's recall occurred after the end of the *second* day of learning. This timeline extended past the time when recall is often initially assessed, at the end of the first (or only) learning session. In addition, this timeline provided an opportunity for consolidation—the process by which recently learned material becomes stabilized and integrated with information in long-term memory. The sleep that occurred between the first and second day likely promoted this consolidation (Davis et al., 2009; Schimke et al., 2021). It is true that there are similarities between the effects of spaced retrieval and those of consolidation because both involve hippocampal-to-neocortical communication (Antony et al., 2017). Nevertheless, the fact that initial testing did not occur until the second day could explain why spaced retrieval benefits were already apparent on the first occasion recall was tested. It is possible that if we had administered a recall test at the end of the first day and then again (as we did) 1 week later, differences between conditions might have been small initially and large (as was observed) at the 1-week point.

### Group Differences

The main effects for group seen for the word form recall and recognition tests were not qualified by any interactions. On these two tasks, the children with TD were simply more capable. The absence of a group difference on the meaning recall test has precedent. Indeed, the same finding was seen in the study on which the present book reading adaptation was based (Leonard, Karpicke, et al., 2019). Again, we cannot rule out the possibility that our meaning task was too simple.

### Inflecting the Novel Words

In previous studies, children with DLD have shown certain kinds of flexibility in their use of recently learned novel words. For example, they have shown the ability to apply a novel adjective to referents with the same attribute that had never been seen before (Leonard, Deevy, et al., 2019). However, the only assessments of these children's ability to change the morphology of the novel words have come from the studies of novel verbs. In those studies, the children with DLD showed very limited flexibility in changing correctly recalled bare stem verbs to the same verbs inflected with –*ing* and vice versa, in contrast to their same-age peers (Leonard et al., 2023, 2024). A decidedly different result emerged in the present study using the noun inflection –*s*. Although only children with DLD participated in this task, their tendency to add the inflection to the novel words they remembered in the first place was quite strong (89% for words in the RSR condition and 96% for words in the RS condition). We interpret these findings to mean that it may not be a general morphological inflexibility in these children but rather a particular problem in modulating newly learned verbs given that the changes in verb forms are based on grammatical aspect and finiteness dictated by grammatical structure. Changes from noun singular to noun plural, in contrast, are more dependent on semantic factors than on structure.

### Remaining Questions

There are additional manipulations that might have enabled the children to learn even more novel words than we observed. For example, if our meanings were more elaborate, such as providing a definition as well as what the object “liked,” gains might have been greater (e.g., Justice et al., 2005). In addition, although the questions used as retrieval prompts represented one useful dialogic device, there are others, such as off-script comments, that might have provided the children with a richer experience with each novel word (Ard & Beverly, 2004).

We also do not know exactly how the novel words and referents interacted with the story line and main characters. We deliberately kept the referents as merely things seen along the way without changing the direction or the behavior of the characters. In contrast, many words in books that are intended to be learned play a much more central role in the story. We do not yet have evidence to argue that we can replicate our current findings if we integrated the referents of the novel words more tightly within the story.

We also have very little understanding of how words learned during shared book reading might generalize. In the laboratory setting, RSR advantages continue to hold when novel adjectives must be applied to objects never before associated with the new attribute (Leonard, Deevy, et al., 2019). However, whether the beneficial effects of RSR are maintained when such generalization occurs within shared book reading has not yet been put to the test.

Finally, we must acknowledge the fact that the facilitative effects of spaced retrieval that we have uncovered thus far have been confined to the mapping problem—the child's task of mapping a new (novel) word to a referent. As insightfully explained by Wojcik et al. (2022), this is but one aspect of word learning and ignores other sources of word learning we all rely on, such as relations among words, the syntactic structure housing new words, and the pragmatic context. How retrieval practice during word learning might function beyond acts of ostensive labeling (as in our word form recall task) and referent selection (as in our recognition task) remains, at this point, territory yet to be explored.

Clearly, then, there are additions and other modifications that might make our retrieval-within-stories procedure more effective. However, we believe that by preserving as many details of the original nonbook procedure as possible, we gained an important first look at how RSR might fare when applied to shared book reading—an everyday activity seen across clinical, educational, and home settings. Thus far, the results are encouraging.

## Data Availability Statement

The data sets used for this study are available from the corresponding author on reasonable request.
